# Nonlinear Growth Dynamics of Neuronal Cells Cultured on Directional Surfaces

**DOI:** 10.3390/biomimetics9040203

**Published:** 2024-03-28

**Authors:** Cristian Staii

**Affiliations:** Department of Physics and Astronomy, Tufts University, Medford, MA 02155, USA; cristian.staii@tufts.edu

**Keywords:** nonlinear dynamics, neuronal growth, neural networks, tissue engineering, stochastic processes, non-equilibrium statistical mechanics

## Abstract

During the development of the nervous system, neuronal cells extend axons and dendrites that form complex neuronal networks, which are essential for transmitting and processing information. Understanding the physical processes that underlie the formation of neuronal networks is essential for gaining a deeper insight into higher-order brain functions such as sensory processing, learning, and memory. In the process of creating networks, axons travel towards other recipient neurons, directed by a combination of internal and external cues that include genetic instructions, biochemical signals, as well as external mechanical and geometrical stimuli. Although there have been significant recent advances, the basic principles governing axonal growth, collective dynamics, and the development of neuronal networks remain poorly understood. In this paper, we present a detailed analysis of nonlinear dynamics for axonal growth on surfaces with periodic geometrical patterns. We show that axonal growth on these surfaces is described by nonlinear Langevin equations with speed-dependent deterministic terms and gaussian stochastic noise. This theoretical model yields a comprehensive description of axonal growth at both intermediate and long time scales (tens of hours after cell plating), and predicts key dynamical parameters, such as speed and angular correlation functions, axonal mean squared lengths, and diffusion (cell motility) coefficients. We use this model to perform simulations of axonal trajectories on the growth surfaces, in turn demonstrating very good agreement between simulated growth and the experimental results. These results provide important insights into the current understanding of the dynamical behavior of neurons, the self-wiring of the nervous system, as well as for designing innovative biomimetic neural network models.

## 1. Introduction

Neuronal cells serve as the fundamental functional units of the brain and play an essential role in the transmission of electrical and chemical signals throughout the nervous system. The basic morphology of a neuronal cell consists of a cell body (soma), a long process (axon), and several shorter processes (dendrites). In the course of brain development, neurons dynamically extend (grow) their axons, spanning lengths from several tens to hundreds of soma diameters, to connect with specific dendrites of other neurons, thereby establishing complex neuronal networks. This intricate process is vital for the formation and function of the nervous system and has profound implications for motor and cognitive functions such as automatic reflexes, learning, attention, and memory. Axonal growth is controlled by the growth cone, a highly motile structure located at the forefront of the axon, which is responsible for sensing biochemical, mechanical, and topographical cues from the surrounding environment as well as from other cells.

The past few decades have seen remarkable advancements in our understanding of the molecular and cellular mechanisms underlying axonal growth [1,2,3,4,5,6,7]. It is now known that axonal guidance is influenced by a variety of factors, including diffusing chemical gradients (Neurotrophins, nerve growth factors, Netrins, Slits, Semaphorins), substrate-bound biochemical cues (Ephrins, extracellular matrix, and cell adhesion molecules), as well as assistance from glial or Schwann cells [1,2,3,4,5,6,7,8]. The interaction of growth cones with these chemical cues can result in either attraction or repulsion, a phenomenon intricately connected to the dynamics of the growth cone’s cytoskeleton [1,2,3,4,9,10]. Furthermore, axonal elongation is heavily influenced by the interplay between the axonal biomechanical properties and the mechanics and geometry of its surrounding environment [7]. The structural integrity and flexibility of axons, provided by their elaborate cytoskeletal network of microtubules and actin filaments, allow for complex mechanical deformations. These biomechanical structures are not passive; growth cones actively generate traction forces, influencing their interaction with the extracellular matrix and, consequently, the direction and speed of axonal growth [10,11,12,13,14].

The latest developments in the fields of microfabrication and microfluidics have opened new avenues for studying neuronal growth in controlled in vitro environments [15,16,17,18]. By manipulating biochemical, mechanical, and geometrical stimuli, researchers have gained critical insights into how external cues affect axonal elongation and alignment. For instance, research has revealed that altering the stiffness of the substrate significantly impacts the axonal growth [11]. Furthermore, it has been demonstrated that periodic geometric patterns on the growth surface as well as asymmetric microfluidic channels not only enhance the extension of axons, but also control their growth direction and promote directional alignment [16,17,18]. In our previous work, we have investigated neuronal growth on poly-D-lysine-coated polydimethylsiloxane (PDMS) substrates with periodic parallel ridge micropatterns [19,20,21,22,23,24,25]. Our studies have demonstrated that axons align parallel to these surface patterns, due to the emergence of a “deterministic torque” induced by the cell–surface interactions, and that axonal dynamics are controlled by a feedback mechanism, which can be modified by the chemical treatment of the cell [23,24]. We have also measured axonal speeds, angular distributions, velocity and angular correlation functions, diffusion (cell motility) coefficients, as well as cell–surface interaction forces and the axonal bending modulus for neuronal growth on these substrates [10,19,20,21,22,23,24,25].

Besides their importance for understanding the basic mechanisms that govern axonal growth, these studies also have substantial practical implications, especially in the fields of nerve repair and tissue engineering. For example, in bioengineering neuroprosthetic devices, the goal is to recreate environments that foster axonal outgrowth and simulate physiological conditions found in vivo. Such endeavors are crucial in the development of new bio-inspired therapeutic approaches for nerve injuries and disorders affecting the nervous system [26,27]. Additionally, acquiring a comprehensive understanding of the processes underlying neuronal growth will stimulate the development of novel biomimetic artificial neural networks that emulate critical functions of the brain. However, despite recent significant advances, many ongoing challenges persist in our current understanding of axonal growth and the formation of neuronal circuits. These include the quantitative relationships between various biochemical and biophysical factors, as well as the biomechanical responses of neurons to external cues, the generation of traction forces, and the specifics of neuron–substrate interactions.

In addition to experimental work, the mathematical modeling of neuronal growth has been a focus of extensive research, involving a variety of approaches and models to understand this complex biological process [28,29]. Axonal growth is fundamentally a product of the combined effects of deterministic and random elements influencing the motility of the growth cone. An example of a deterministic factor is the cell-substrate traction forces generated during axonal elongation [10,11,12,13,14]. On the other hand, random influences include the polymerization of cytoskeletal structures like actin filaments and microtubules, cellular signaling processes, the detection of chemical gradients and biomolecules at low concentrations, intracellular biochemical reactions, and the development of lamellipodia and filopodia within the growth cone [1,2,3,4,5,6,7]. Due to this combination of deterministic and random elements, predicting the growth path of individual axons is very challenging. However, it is possible to describe the general characteristics of ensembles of neurons using probability functions that obey a set of precisely defined stochastic differential equations [28,29,30,31,32,33].

A common method for modelling the interplay between the deterministic and random processes that drive neuronal growth is based on using Langevin and Fokker–Planck equations. Solutions to these equations provide probability distributions for the various parameters that describe axonal dynamics, thus allowing predictions regarding neuronal network formation under varying external conditions. For example, pioneering work by Hentschel and van Ooyen used these models to explain how a combination of attractant and repellent factors influences axon bundling and guidance [30]. Maskery and collaborators employed Langevin simulations to predict the minimum detectable chemical gradients for specific experimental configurations [31], while the Drew group solved the Fokker–Planck equation to describe growth cone paths for simple environments in the absence of external cues [32]. Betz and colleagues used the Fokker–Planck framework to quantify the stochastic fluctuations in the lamellipodia of the growth cone. Their work highlighted that the observed bimodal dynamics of the growth cone emerges from the stochastic polymerization of actin filaments inside the cell [33].

In our previous work, we employed Langevin and Fokker–Planck equations to construct a comprehensive theoretical framework for predicting the behavior of growth cones and explaining how various external signals affect neuronal growth [19,20,21,22,23,24,25]. For instance, our earlier findings indicated that the growth patterns of neuronal cells grown on flat glass surfaces coated with poly-D-lysine (PDL) are described by linear Langevin equations with stochastic white noise, which lead to a collective regulatory mechanism for the axonal growth speeds on these substrates [20]. Additionally, we have utilized Langevin and Fokker–Planck equations to analyze axonal growth and calculate the diffusion coefficient of growth cones on surfaces featuring ratchet-like structures which are made of asymmetrically tilted nanorods [19]. Through several studies, we have also emphasized that periodic micropatterns on the growth surfaces significantly influence the direction of axonal growth [21,22,23,24,25]. We have shown that the axonal growth on these patterned surfaces is best understood by considering the interplay between stochastic signals and the deterministic factors represented by geometrical and mechanical guidance cues. Our previous findings also indicate that axonal growth is governed by feedback mechanisms wherein the growth cone continually senses external signals, adjusting its path in response to these environmental stimuli [24].

In this paper, we combine experimental observations with theoretical analysis to incorporate nonlinear processes in the stochastic models of axonal growth. We build on our recent work where we have shown that periodic geometrical features generate a drift term applied to the growth cone, and the stochastic components produce a random walk motion along the axonal growth direction [25]. Here, we demonstrate that the drift term is inherently nonlinear and could lead to a broad range of dynamical behaviors in neuronal growth, including pattern formation, alternating regimes of motion, and anomalous diffusion. We experimentally measure angular and speed distributions as well as correlation functions, using this data to predict axonal mean squared lengths and diffusion (cell motility) coefficients. We also use the nonlinear model to simulate axonal growth and find excellent agreement with the experimental data. Our findings have significant implications not only for enhancing the current understanding of neuronal growth, but also for the creation of innovative bioinspired neural networks. Additionally, they could contribute to the development of novel bioengineered substrates that promote nerve repair and regeneration.

## 2. Materials and Methods

In this research, cortical neurons from 18-day-old rat embryos were utilized. The brain tissue-handling procedure was approved by Tufts University Institutional Animal Care Use Committee, and it is in agreement with the National Institutes of Health’s guidelines. We have used established methods, detailed in prior publications [9,10,19,20,21,22,23,24,25], for cell dissociation and culture. Our team’s earlier immunostaining experiments have confirmed a high purity of neurons in these cultures [9]. Neuronal cells were cultured on micropatterned polydimethylsiloxane (PDMS) surfaces, which were treated with poly-D-lysine (PDL), maintaining a density of 4000 cells per square centimeter. As noted in our previous studies, neurons grown at relatively low densities (ranging from 3000 to 7000 cells per square centimeter) tend to develop long axons, suitable for studying growth dynamics on surfaces with varied external stimuli [19,20,21,22,23,24,25].

**Figure 1:**
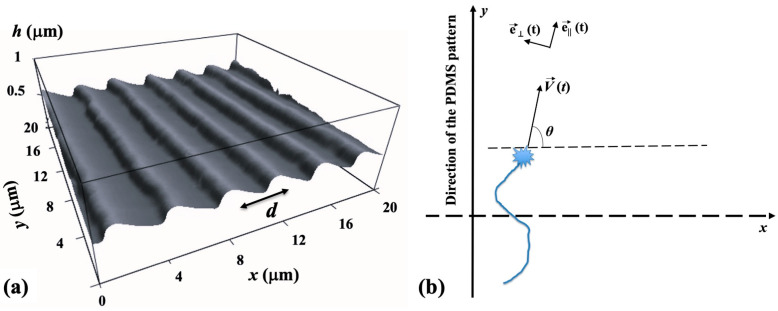
(**a**) Example of an AFM topography image of a PDL-coated PDMS surface with periodic micropatterns. The figure demonstrates that the micropatterns are periodic in the *x* direction with the spatial period *d* and have a constant maximum height *h* of approximately 0.5 μm. (**b**) Schematic of the coordinate system. The blue drawing shows the schematic of an axon and the growth cone. The *y* axis is defined as the axis parallel to the direction of the PDMS patterns. The growth angle *θ* is defined as the angle between the axonal velocity and the *x* axis at a given time *t*. The figure inset shows the parallel and perpendicular directions of motion, represented by the pair of time-dependent unit vectors $\left({\vec{e}}_{\left|\right|}\left(t\right),{\vec{e}}_{⊥}(t)\right)$.

The periodic micropatterns on the PDMS surfaces are composed of parallel ridges separated by grooves (Figure 1a). These patterns were created using a straightforward imprinting technique, which involved pressing diffraction grids onto the PDMS substrates [23]. The gap between the two adjacent ridges on these surfaces is referred to as the pattern spatial period *d*. An atomic force microscope (AFM) image showing these micropatterns is displayed in Figure 1a.

The micropatterned surfaces are spin-coated with a PDL (Sigma-Aldrich, St. Louis, MO, USA) solution with 0.1 mg/mL concentration. AFM images of the growth substrates and fluorescence images of growing neurons have been acquired using an MFP3D AFM that includes a BioHeater closed fluid cell and an inverted Nikon Eclipse Ti optical microscope (Micro Video Instruments, Avon, MA, USA). Fluorescence images have been acquired using a standard Fluorescein isothiocyanate -FITC filter as follows: excitation—495 nm and emission—521 nm. Further details on substrate preparation, microfabrication techniques, as well as fluorescence and AFM imaging are available in our previous Biomimetics publication [23].

**Data analysis.** The motion of the growth cones has been monitored using ImageJ (version 1.53h 04, National Institute of Health, Bethesda, MS, USA). The change in the spatial positions of the growth cone with time was measured using fluorescence microscopy by tracking the change in the center of the growth cone position every Δ*t* = 5 min for a total period of 30 min for the images taken at the following time intervals following neuron culture: *t_culture_* = 10, 15, 20, 25, 30, 35, 40, 45, and 50 h. The time interval Δ*t* = 5 min between measurements was selected so that the magnitude of the displacement $\Delta \vec{r}$ of the growth cone is greater than the experimental precision of our measurement (~0.1 μm), and that the ratio $\Delta \vec{r}/\Delta t$ is close to the instantaneous velocity $\vec{V}$ of the growth cone. The angle *θ* is measured relative to the *x* axis (*θ* as well as the *x* and *y* axes are defined in Figure 1b). To determine the speed distributions, the range of growth cone speeds at each time point was divided into intervals of equal size. The speed correlation function was obtained with the following formula [21,22]:

(1) $$\langle V\left(t_{1}\right)\cdot V\left(t_{2}\right)\rangle =\frac{1}{N}\cdot \sum_{i=1}^{N}\left[V_{i}\left(t_{1}\right)\cdot V_{i}\left(t_{2}\right)\right]\;\;\;$$

where *N* is the total number of growth cones and $V_{i}\left(t_{1}\right),\;{\;V}_{i}\left(t_{2}\right)$ represent the speeds for the *i*th growth cone at times *t*_1_ and *t*_2_, respectively.

**Numerical Simulations**. We perform simulations of growth cone trajectories using the stochastic Euler–Maruyama method with N steps [32,34,35,36]. With this method, the position of the growth cone is parametrized by the arclength *s* from the axon’s initial position. The turning angle at each step, representing the randomness in the axon steering, is determined from the stochastic component of an uncorrelated Wiener process [23,25]. The simulated growth velocities are obtained from the change in position of the growth cone at each step [23,24,34,35,36].

## 3. Results

### 3.1. Experimental Results of Axonal Growth on PDMS Substrates

**Figure 2:**
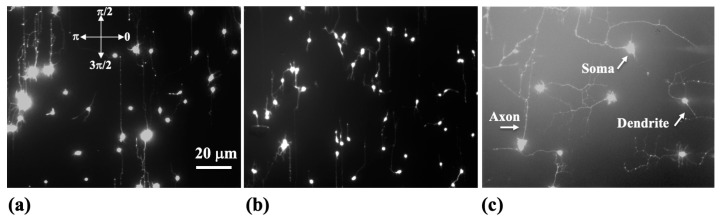
Fluorescence (Tubulin Tracker Green) images showing examples of axonal growth images for cortical neurons cultured on PDL-coated PDMS surfaces. (**a**,**b**) Examples of growth for neurons cultured on micropatterned PDMS substrates with *d* = 3 μm in (**a**), and *d* = 5 μm in (**b**). The directions corresponding to the growth angle θ= 0, π/2, π, and 3π/2 are shown in (**a**). The micropatterns promote directional growth along the *y* axis. (**c**) Example of axonal growth on a flat PDMS surface (without micropatterns). The figure also displays the main structural components of a neuron: soma, axons, and dendrites. Cortical neurons typically grow one long axon and several shorter dendrites. The growth cone is located at the tip of the axon. All images are captured at *t_culture_* = 25 h after cell plating, corresponding to the observation time *t* = 15 h (see main text). The scale bar shown in (**a**) is the same for all images.

In this work, cortical neurons are cultured on PDL-coated PDMS surfaces with the two different pattern spatial periods *d* = 3 μm and *d* = 5 μm. Axonal growth on these surfaces is measured at different time points *t_culture_* after cell plating. Figure 2a,b show examples of images of axonal growth taken at *t_culture_* = 25 h on substrates with *d* = 3 μm (Figure 2a) and *d* = 5 μm (Figure 2b). For comparison, Figure 2c shows an example of axonal growth on a flat PDMS surface without micropatterns.

**Figure 3:**
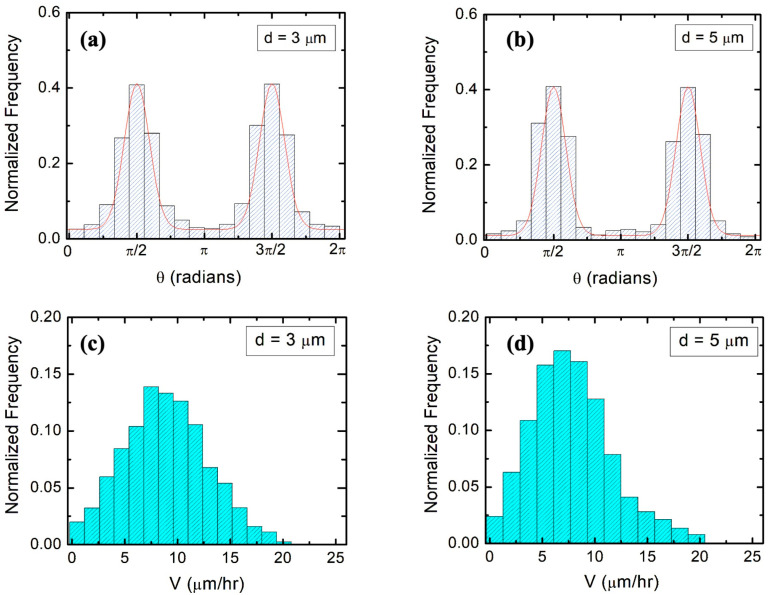
(**a**,**b**) Examples of normalized experimental angular distributions for axonal growth for neurons cultured on micropatterned PDMS surfaces with pattern spatial period *d* = 3 μm in (**a**), and *d* = 5 μm in (**b**). The vertical axis (labeled Normalized Frequency) represents the ratio between the number of axonal segments growing in a given direction and the total number N of axon segments. Experimental data are obtained from measurements on N = 972 different axon segments for neurons cultured on surfaces with *d* = 3 μm, and for N = 920 different axon segments for neurons cultured on surfaces with *d* = 5 μm, respectively. Each axonal segment is of 20 μm in length (see section on Data Analysis). The continuous red curves in (**a**,**b**) are fits for the data with Equation (2). The data show that the axons display strong directional alignment along the surface patterns (peaks at θ= π/2 and θ= 3π/2), with a high degree of alignment given by the variance of the distributions. (**c**,**d**) Examples of normalized speed distributions for growth cones measured on micropatterned PDMS surfaces for neurons cultured on surfaces with pattern spatial period *d* = 3 μm in (**c**), and *d* = 5 μm in (**d**). All distributions show data collected at *t_culture_* = 25 h after neuron plating, corresponding to an observation time *t* = 15 h (see main text).

In our previous research, we have established that (1) axons of neurons cultured on micropatterned PDMS surfaces grow predominantly along the pattern directions; (2) axonal alignment along the surface pattern increases with time; and (3) the highest degree of axonal alignment for a given time occurs when the pattern spatial period *d* matches the growth cone’s linear size, that is, when *d* is in the range 3–5 μm [21,22,23]. We have also shown that axons tend to grow along the ridges of the patterns, with relatively few axons crossing between the neighboring ridges [21]. Figure 3a and b show examples of angular distributions measured at *t_culture_* = 25 h for neuronal growth on surfaces with *d* = 3 μm (Figure 3a) and *d* = 5 μm (Figure 3b). Figure 3c,d show experimentally measured speed distributions for these samples (these experimental distributions will be discussed in the next section).

In previous work, we have demonstrated that the angular distribution of axons is described by a Fokker–Planck equation with the following stationary solution [23]:

(2) $$p(\theta )=A\cdot exp\left(\frac{\gamma_{\theta }}{D_{\theta }}\cdot |sin\;(\theta )|\right)$$

where *A* is a normalization constant, $\gamma_{\theta \;}$ is the magnitude of a “deterministic torque” which tends to align the axon with the direction of the surface micropattern, and *D_θ_* represents the angular diffusion coefficient. Here, we define the effective angular correlation rate as $k_{\theta }=\;\gamma_{\theta }/D_{\theta }$. Equation (2) shows that the axons exhibit alignment along the direction of the micropatterns (angular distributions centered at θ=π/2 and θ= 3π/2). By fitting the angular distributions in Figure 3a,b with Equation (2) (continuous red curves in the figures), we obtain the following values for the angular correlation rates [23]: $k_{1\theta }=\left(1.7\;\pm 0.02\right)$ h^−1^ (for *d* = 3 μm, Figure 3a), and $k_{2\theta }=(2.2\;\pm 0.2)$ h^−1^ (for *d* = 5 μm, Figure 3b). Furthermore, from the experimentally measured speed distributions (Figure 3c,d), we extract the values for average speed $\langle V\rangle$ and variance $\sigma_{V}^{2}=\langle V^{2}\rangle -{\langle V\rangle }^{2}$. We obtain $\langle V\rangle =8.7$ μm/h and $\sigma_{V}^{2}=16$ μm^2^/h^2^ (for *d* = 3 μm), and $\langle V\rangle =7.2$ μm/h, and $\sigma_{V}^{2}=12.25$ μm^2^/h^2^ (for *d* = 5 μm).

### 3.2. Theoretical Modelling of Axonal Dynamics Based on Nonlinear Langevin Equations

In reference [22], we have experimentally determined that the axonal growth on micropatterned PDMS substrates is governed by two nonlinear Langevin equations of motion as follows:

(3) $$\;\;\;\;\;\;\;\;\;\;\;\;\;\;\;\;\;{\left(\frac{d\vec{V}}{dt}\right)}_{\left|\right|}=a_{0}|\sin\theta |-\gamma_{1}\cdot V-\gamma_{2}\cdot V^{2}+\Gamma_{\left|\right|}\left(t\right)\;\;\;\;\;\;\;\;\;\;\;\;$$

(4) $${\left(\frac{d\vec{V}}{dt}\right)}_{⊥}=a_{1}\cos\theta +\Gamma_{⊥}\left(t\right)\;\;$$

In the above equations, ${\left(d\vec{V}/dt\right)}_{\left|\right|}$ and ${\left(d\vec{V}/dt\right)}_{⊥}$ are, respectively, the parallel and perpendicular components of the growth cone acceleration, and *θ* represents the growth angle with respect to the *x* axis (the *x* axis as well as the parallel and perpendicular directions of motion, represented by the pair of time dependent unit vectors $\left({\vec{e}}_{\left|\right|}\left(t\right),{\vec{e}}_{⊥}(t)\right)$ are defined in Figure 1b). The growth cone velocity is given by $\vec{V}=V\cdot {\vec{e}}_{\left|\right|}$, where *V* is the speed of the growth cone. The parameters *a*_0_, *a*_1,_ *γ*_1_, *γ*_2_ in Equations (3) and (4) are velocity-independent parameters that depend on the pattern spatial period *d* of the PDMS growth surface. We have shown that all these parameters can be obtained experimentally by analyzing the spatial and temporal evolutions of axonal growth [21,22,23]. In addition, the stochastic contributions for parallel and perpendicular growth, $\Gamma_{\left|\right|}$ and $\Gamma_{⊥}$ in Equations (3) and (4), satisfy the conditions for gaussian white noise with zero mean, characteristic to uncorrelated Wiener processes [22] as follows:

(5) $$\langle \Gamma_{\left|\right|}(t)\rangle =0\;\;\;$$

(6) $$\langle \Gamma_{\left|\right|}(t_{1})\Gamma_{\left|\right|}(t_{2})\rangle =\sigma^{2}\cdot \delta \left(t_{1}-t_{2}\right)$$

with similar expressions for $\Gamma_{⊥}$. In Equations (5) and (6), σ is a term that quantifies the strength of the noise (the variance of the stochastic distribution) and $\delta \left(t_{1}-t_{2}\right)$ is the Dirac delta function.

In our recent work [25], we have shown that the perpendicular component of the acceleration (Equation (4)) plays the role of a deterministic torque that aligns the growth cone motion in the direction of the micropattern. The experiments demonstrate that the perpendicular component of the acceleration has maximum values at the beginning of the axonal growth [25]. Indeed, if the axons elongate in directions perpendicular to the micropattern (i.e., long the *x* axis in Figure 1b), we have maximum ${\left(dV/dt\right)}_{⊥}$ for θ≈0, as predicted by Equation (4). As a result of this process, as time increases, axons tend to continue their growth along the micropatterns (i.e., the *y* direction, characterized by $\theta \approx \frac{\pi }{2}$ or $\theta \approx \frac{3\pi }{2}$, see Figure 1b). Therefore, at later times, the deterministic torque in Equation (4) is negligible, $a_{1}\cos\theta \;\approx 0$, and the axons display a directional growth, oriented in average along the *y* direction. Moreover, the stochastic terms generate fluctuations along this average growth direction. We have experimentally demonstrated that these conditions are met for growth times *t_culture_* in the interval 10–50 h [22,23,24,25]. For larger times (*t_culture_* > 50 h), most axons establish connections with other neuronal cells and the growth phase ends. In this paper, we define the observation time *t* as *t* = *t_culture_* − 10 h, and measure axonal growth in the time interval 0≤t≤40 h, in time increments of 5 h (this corresponds to times *t_culture_* = 10–50 h after plating).

In our previous work [25], we have analyzed axonal growth along the direction of the pattern for PDMS surfaces with relatively large values of the pattern spatial period as follows: d ≥7 μm. Experimental data show that in this case, $\gamma_{2}\cdot V^{2}\ll \gamma_{1}V$ for typical growth speeds of the order of 10 μm/h. We have demonstrated that under this condition, the axonal dynamics on the micropatterned PDMS substrates is characterized by a biased random walk, in which the surface geometry imparts a constant drift term to the growth cone, and the stochastic components lead to a diffusive motion around the average growth direction. The drift-diffusion process is marked by an increase in the axonal mean squared velocity in the direction of the micropattern, with a cell motility (diffusion) coefficient $D\approx 21\;{\mu m}^{2}/h$ [25]. We have also experimentally measured the parameters *γ*_1_ and *γ*_2_ in Equation (3), finding that $\gamma_{1}\;\approx 0.1\;h^{-1}$ is approximately constant, independent of *d*, whereas the parameter *γ*_2_ increases with the decreasing spatial period *d* [22]. In particular, we have reported that $\gamma_{2}\;\approx 0.8\;{\mu m}^{-1}$ (for *d* = 3 μm) and $\gamma_{2}\;\approx 0.7\;{\mu m}^{-1}$ (for *d* = 5 μm) [22], which implies that for these spatial periods and for growth cone speeds of the order of ~10 μm/h observed in our experiments (Figure 3c,d), both the linear and the quadratic terms in Equation (3) are important.

In this paper, we analyze the axonal dynamics on surfaces with *d* = 3 μm and *d* = 5 μm, and for culture times $t_{culture}\;\geq 10\;h$ (i.e., observation times t ≥0 h). Under these conditions, as discussed above, the deterministic torque is negligible, $a_{1}\cos\theta \;\approx 0$, and the axonal velocity is independent of the stochastic variations in the growth angle θ. In this case, the general theoretical model describing the axonal dynamics is defined by the following pair of nonlinear stochastic differential equations for the axonal speed *V* and growth angle θ as follows:

(7) $$\;\frac{dV}{dt}=-\gamma (V)\cdot V+\Gamma_{V}\left(t\right)$$

(8) $$\frac{d\theta }{dt}=\Gamma_{\theta }\left(t\right)\;$$

In the above equations, γ(V) is a speed-dependent friction function, and $\Gamma_{V}\left(t\right)$, $\Gamma_{\theta }\left(t\right)$ are stochastic terms for speed and angle, respectively, satisfying gaussian white noise conditions similar to Equations (5) and (6) above. We note that with the definition of velocity vector $\vec{V}=V(t)\cdot {\vec{e}}_{\left|\right|}(t)$ (Figure 1b) and for the particular case of a quadratic friction function γ(V) and negligible deterministic torque, the general nonlinear Equations (7) and (8) reduce to Equations (3) and (4). The non-linear Equations (7) and (8) are the starting point for our current analysis. We use this model to calculate the dynamical parameters of axonal growth and compare these predictions with experimental results.

Since *V* and θ are independent variables in the above equations, the joint time-dependent probability distribution can be decomposed as $P\left(V,\theta ,t\right)=P_{V}\left(V,t\right)\cdot P_{\theta }\left(\theta ,t\right).$ The expectation value for the mean squared displacement, i.e., the axonal mean squared length $\langle L^{2}\left(t\right)\rangle$ of the growth cone, is then given by the following equation [25,37,38]:

(9) $$L^{2}\left(t\right)\equiv \langle {\vec{r}}^{2}\left(t\right)\rangle =\int_{0}^{t}{dt}_{1}\int_{0}^{t_{1}}dt_{2}\langle \vec{V}\left(0\right)\cdot \vec{V}\left(t_{2}\right)\rangle \;\;=\;\int_{0}^{t}{dt}_{1}\int_{0}^{t_{1}}dt_{2}\langle V\left(0\right)\cdot V\left(t_{2}\right)\rangle \cdot \langle {\vec{e}}_{\left|\right|}(0)\cdot {\vec{e}}_{\left|\right|}(t_{2})\rangle$$

**Figure 4:**
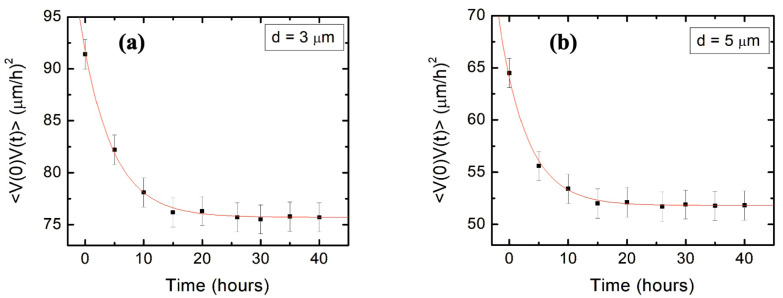
(**a**) Variation of the speed correlation function with time for neurons cultured on micropatterned PDMS surfaces with pattern spatial period *d* = 3 μm in (**a**) and *d* = 5 μm in (**b**). The data points indicate the speed correlations determined experimentally at different times. Each data point in (**a**,**b**) was acquired by measuring between N = 65 and N = 93 growth cones. Error bars indicate the standard error of the mean. The continuous red curves are fits for the data with Equation (10), which give the speed correlation rate $k_{V}$ (see text).

Quite generally, the nonlinear model described by Equations (7) and (8) results in exponential decays with time of the speed and angular correlation functions as follows [37,38]:

(10) $$\langle V\left(0\right)\cdot V\left(t\right)\rangle ={\langle V\rangle }^{2}+\sigma_{V}^{2}\cdot e^{-k_{V}t}$$

(11) $$\langle {\vec{e}}_{\left|\right|}(0)\cdot {\vec{e}}_{\left|\right|}(t_{2})\rangle =e^{-k_{\theta }t}$$

where $\sigma_{V}^{2}=\langle V^{2}\rangle -{\langle V\rangle }^{2}$, and $k_{V}$ and $k_{\theta }$ are the speed and angular correlations decay rates. Here, the values for ${\langle V\rangle }^{2}$, $\sigma_{V}^{2}$, $k_{\theta }$ are obtained from the speed and angular distributions shown in Figure 3 (as discussed in Section 3.1). Figure 4 shows the experimentally measured speed correlation functions for *d* = 3 μm (Figure 4a) and *d* = 5 μm (Figure 4b). From the fit of the data in Figure 4 with Equation (10), we obtain the following values for the speed correlation decay rates (fit parameter): $k_{V}=0.19$ h^−1^ (for *d* = 3 μm) and $k_{V}=0.21$ h^−1^ (for *d* = 5 μm).

Inserting Equations (10) and (11) into Equation (9), we obtain the following expression for the axonal mean square length:

(12) $$\;\;\;\langle L^{2}\left(t\right)\rangle =2\frac{{\langle V\rangle }^{2}}{k_{\theta }}\cdot \left[t+\frac{1}{k_{\theta }}\cdot \left(e^{-k_{\theta }t}-1\right)\right]+\;2\frac{\sigma_{V}^{2}}{k_{\theta }+k_{V}}\cdot \left[t+\frac{1}{k_{\theta }+k_{V}}\cdot \left(e^{-\left(k_{\theta }+k_{V}\right)t}-1\right)\right]$$

**Figure 5:**
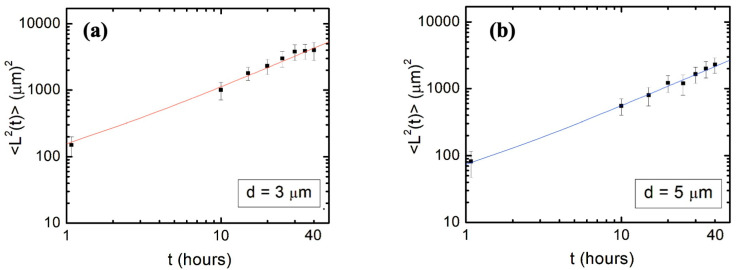
(**a**,**b**) Log–log plots of the axonal mean squared length vs. time for neurons cultured on micropatterned PDMS surfaces with pattern spatial period *d* = 3 μm in (**a**) and *d* = 5 μm in (**b**). Data points indicate mean square lengths for axons obtained from experiment. Each data point in (**a**,**b**) was acquired by measuring between N = 65 and N = 93 axons. Error bars indicate the standard error of the mean for each data set. The continuous red (**a**) and blue (**b**) curves represent the plots of Equation (12) for the two different types of surfaces, without any additional free parameters.

Figure 5 shows the experimental data for the axonal mean squared length as well as the plots of Equation (12) (continuous curves) for axonal growth on surfaces with for *d* = 3 μm (Figure 5a), and *d* = 5 μm (Figure 5b). All parameters appearing in Equation (12) have been measured from the data fit in Figure 3 and Figure 4, as described above. Therefore, the red curve in Figure 5a (respectively, the blue curve in Figure 5b) represents plots of Equation (12) without the introduction of any additional free parameters. We conclude that the theoretical prediction of the nonlinear model given by Equations (7)–(12) shows remarkable agreement with the experimental data for the axonal mean squared length.

### 3.3. Diffusion Coefficient and Simulations of Axonal Dynamics

The nonlinear model introduced in the previous section predicts several different scaling regimes for the axonal mean squared length with time. In particular, for short times *t*, we have $\langle L^{2}\left(t\right)\rangle \;\sim {\;t}^{2}$, commonly referred to as ballistic regime [37,38]. In the limit of large times, we obtain a diffusive regime characterized by $\langle L^{2}\left(t\right)\rangle \;\sim \;t$. The cross over between the two regimes occurs at a characteristic time $\tau =\frac{1}{k_{\theta }}\approx 5$ h (i.e., at 15 h after the cell culture). In the limiting diffusion regime, we can define a diffusion coefficient that describes the diffusive spread around the mean direction of motion (micropattern direction) as follows [25,39]:

(13) $$\;D=\int_{0}^{\infty }\left(\langle V\left(0\right)\cdot V\left(t\right)\rangle -{\langle V(t)\rangle }^{2}\right)dt$$

From Equations (12) and (13) we obtain for large times t≫τ the following expression for *D*:

(14) $$\;\;D=\frac{{\langle V\rangle }^{2}}{k_{\theta }}+\;\frac{\sigma_{V}^{2}}{k_{\theta }+k_{V}}$$

This represents an effective diffusion coefficient which characterizes the limiting diffusion regime. Inserting the parameters measured for growth on surfaces with different *d* values (previous section), we obtain $D\approx 53\;{\mu m}^{2}/h$ (for *d* = 3 μm), and $D\approx 28\;{\mu m}^{2}/h$ (for *d* = 5 μm). The lower value of *D* for growth on surfaces with *d* = 5 μm is consistent with smaller axonal mean squared lengths measured on these surfaces compared to the corresponding values for growth on surfaces with *d* = 5 μm (see Figure 5).

Finally, we perform simulations of the growth dynamics described by Equations (7) and (8), employing the following particular form of the friction function determined in our previous experiments [22]:

$$\gamma \left(V\right)=\gamma_{1}\cdot V-\gamma_{2}\cdot V^{2},\;\mathrm{with}\;\gamma_{1}\;\approx 0.1\;h^{-1}\;(\mathrm{constant}),\;\mathrm{and},\;\mathrm{respectively}\;\gamma_{2}\;\approx 0.8\;{\mu m}^{-1}(\mathrm{for}\;d\;=\;3\;\mu m)\;\mathrm{and}\;\gamma_{2}\;\approx 0.7\;{\mu m}^{-1}\;(\mathrm{for}\;d\;=\;5\;\mu m).$$

**Figure 6:**
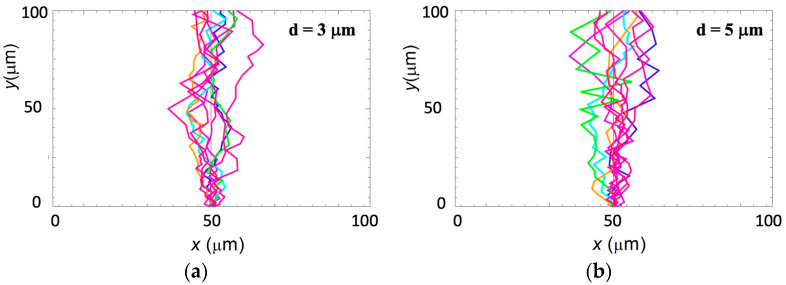
(**a**,**b**) Examples of simulated axonal trajectories for neuronal cells grown on micropatterned PDMS surfaces with pattern spatial period *d* = 3 μm in (**a**) and *d* = 5 μm in (**b**). The simulations are performed by using the values of the growth parameters obtained from the experiment (see main text).

Figure 6a shows examples of simulation results for growth on surfaces with *d* = 3 μm. Simulations corresponding to axonal growth on surfaces with *d* = 5 μm are shown in Figure 5b. The angular and speed distributions obtained from these simulations match the experimental data obtained for effective diffusion coefficients as follows: $D_{s}\approx 50\;{\mu m}^{2}/h$ (for *d* = 3 μm) and $D_{s}\approx 30\;{\mu m}^{2}/h$ (for *d* = 5 μm). These values for the simulated diffusion coefficients are in excellent agreement with the analytical values predicted by the nonlinear growth model (Equation (14)).

## 4. Discussion

Axonal dynamics during neuronal development arise from the intricate interplay between deterministic and stochastic stimuli that influence the growth cone. In our work, we have shown that parallel geometrical features micropatterned on PDMS surfaces facilitate directional axonal alignment, and thus constitute the primary deterministic factors that direct neuronal growth on these surfaces. Our previous results demonstrate that axons exhibit their maximum degree of alignment when the spatial periodicity *d* of the geometrical patterns is close to the average linear dimension of the growth cone, that is, for *d* in the range of 3–5 μm [22]. This range is relevant for the periodic physiological scaffolds, such as glial fibers, extracellular matrix protein tracks, and brain foldings, that facilitate neuronal growth in vivo [1,2,3,4,5,6,7]. We have also shown that neuronal growth cones advance on PDMS surfaces by converting external mechanical and topographical stimuli into directional motion through a contact–guidance process, which requires a coordinated regulation of cytoskeleton dynamics, cell adhesion, and membrane processes [24]. We have measured traction forces and stresses generated by the contact–guidance mechanism, as well as changes in the neuron biomechanical properties during growth [10,24].

In this paper, we investigate neuronal growth on PDMS surfaces with *d* = 3 μm and *d* = 5 μm. We demonstrate that axonal dynamics on these substrates are described by a nonlinear stochastic model given by the Langevin Equations (7) and (8). The model incorporates speed-dependent friction function γ(V) and gaussian white noise. This model shows that the motion of the axon on these surfaces has the two following components: (1) an extension along the direction of the PDMS micropatterns (*y* axis in Figure 1b); and (2) random fluctuations around these main growth directions. There are many possible stimuli responsible for the random motion, including the dynamic nature of the growth cone cytoskeleton, which involves the stochastic polymerization of actin filaments and microtubule, stochasticity in the formation of lamellipodia and filopodia, as well as concentration fluctuations of chemoattractants and chemorepellents [1,2,3,4,5,6,7,30,31,32,33]. In our model, this intrinsic stochasticity of axonal growth is characterized by gaussian white noise (Equations (5) and (6)).

We also show that the main parameters that characterize the axonal growth on micropatterned PDMS surfaces with pattern spatial period d≤5 μm are the average speed $\langle V\rangle$ and variance $\sigma_{V}^{2}$ of the speed distributions, as well as the speed and angular correlation decay rates $k_{V}$ and $k_{\theta }$. We obtain these parameters from experimental data (Figure 3 and Figure 4) and use them to calculate the values for axonal mean length square $\langle L^{2}\left(t\right)\rangle$. Figure 5a,b show excellent agreement between the theoretical predictions for $\langle L^{2}\left(t\right)\rangle$ obtained from Equation (12) (continuous red and blue curves) and the experimental measurements for the axonal mean squared length (black dots) on surfaces with *d* = 3 μm and *d* = 5 μm, respectively. We emphasize that the red and blue curves in Figure 5 represent the theoretical predictions for $\langle L^{2}\left(t\right)\rangle$ plotted without any additional adjustable parameters.

We use the nonlinear dynamical model to calculate diffusion (cell motility) coefficients that characterize axonal motion at large values of the growth time *t*. Using the experimentally measured values for the growth parameters, we obtain $D\approx 53\;{\mu m}^{2}/h$ (for *d* = 3 μm) and $D\approx 28\;{\mu m}^{2}/h$ (for *d* = 5 μm). These values for *D* are larger than the typical values $D\approx 20\;{\mu m}^{2}/h$ reported in our previous work for the diffusion coefficient of 1D axonal growth along the direction of the pattern on surfaces with *d* = 7 μm. The increase in the values of *D* obtained here represent a measure of the increase in the axonal random walk superimposed to the overall 1D directional growth. Moreover, the increase in *D* as the surface pattern spatial period *d* is decreasing is consistent with the larger values for the average axonal mean length squared $L^{2}\left(t\right)$ measured at lower *d* (Figure 5).

Stochastic models of neuronal growth provide fundamental insights into the dynamics of growth cones in response to internal and external cues. We find that the stochastic simulations of axonal trajectories match the experimental data for angle and speed distributions of axons, as well as for the measured values for axonal mean lengths squared if the diffusion coefficients are in agreement with the values predicted by the nonlinear model described by Equations (7)–(14). The cross over to the diffusive regime occurs at a characteristic time scale $\tau =1/k_{\theta }\approx 5$ h (i.e., *t_culture_* = 15 h after the cell plating). Furthermore, fluctuations in axonal speed and angular distributions may lead to additional time scales and crossover regimes. For example, Peruani and Morelli showed that if the angular correlations decay much slower than the speed correlations for active Brownian particles, then the mean square displacement (the parameter corresponding to $\langle L^{2}\left(t\right)\rangle$ in our experiments) displays four distinct regimes and three crossovers in between [38]. At low *t*, the particle moves initially in one dimension (1D) along its initial direction of motion. This 1D motion becomes more random over time, leading to the first crossover between ballistic and diffusive motion. As *t* increases and the velocity reaches a stationary state, the particle dynamics are governed only by its mean speed $\langle V\rangle$ along its average direction of motion. In this stage, the particle continues its effectively 1D motion with $\langle L^{2}\left(t\right)\rangle \;\sim {\;t}^{2}$. This results in a second transition from the transient diffusive regime back to a second ballistic one. Ultimately, at large times *t*, a gradual change in the direction of motion causes a third shift towards a diffusive final state.

In this paper, we focus on axonal growth occurring during the later diffusive regime, which takes place at times 15 h < *t_culture_* < 50 h following cell plating. However, in our previous work, we have reported that the additional bias imparted by the substrate geometry introduces another time scale at *t_culture_* > 50 h and a final crossover to superdiffusive motion, which is characterized by a power law increase in axonal mean squared length with time [21]. This behavior could be derived from a nonlinear persistent random walk model, as shown in reference [28]. In future work, we will investigate if the nonlinear dynamical model can predict the decay of the velocity correlation functions and the parameters of the superdiffusive dynamics which have been experimentally observed [21].

Neurons, by their very nature, exhibit a range of dynamic behaviors that are inherently nonlinear, involving feedback mechanisms, threshold effects, and a sensitivity to initial conditions [1,2,3,4,5,6,7,23,24]. This nonlinearity is essential for understanding how neurons grow, adapt, and form functional networks. Researchers continue to integrate diverse approaches to better understand and predict the intricate process of neuronal development. For example, work completed by the Goodhill group introduced feedback loop mechanisms at the level of the growth cone, leading to nonlinear models that predict different growth states based on the dynamics of the cell point contacts [40]. However, the complexity of the biochemical processes involved in neuronal growth has, so far, limited our ability to model the actual biophysical mechanisms.

The robust information processing by the neurons during growth requires both high sensitivity to external cues as well as low sensitivity to random fluctuations in the surrounding microenvironment. These are the basic characteristics of feedback control, which refers to a general class of the regulatory mechanisms used by biological systems to adapt their behavior to changing external conditions [28,41]. Recent work has demonstrated that both positive (signal amplification) and negative (signal inhibtion) feedback signals are key regulatory processes underlying neuronal growth and development [42,43,44,45]. For example, positive feedback loops control axonal formation and elongation through the local activation of neurotrophin receptors and the accumulation of BDNF, NT-3, and Shootin1 proteins [42,43,44]. Negative feedback signals, such as the local depletion of growth factors and the activation of RhoA and Rho-kinase proteins, determine dendrite development and inhibit the formation of multiple axons [45,46]. However, the interplay between these feedback loops, and the many possible roles that they might play in controlling axonal dynamics, in regulating long range extracellular signaling, and in the emergence of collective cell behavior, is largely unexplored. In addition, more sophisticated mathematical models that incorporate feedback control and nonlinear dynamics with the biophysical and biochemical complexities of neuronal growth are just beginning to emerge [29].

Although we do not incorporate feedback directly, our results set the stage for further investigations into axonal growth and for constructing theoretical models that connect nonlinear dynamics with the feedback control mechanisms. Such mechanisms play a crucial role in how cells respond to external stimuli and regulate intracellular processes like cytoskeletal dynamics and the generation of traction forces. In particular, we anticipate that the coupling between feedback control and stochastic growth should give rise to the rich dynamical behavior observed in other systems, including the emergence of multiple stable equilibrium states that the cell can switch between based on the external input, as well as emergent collective behavior, such as swarming, anomalous diffusion, and phase transitions [47,48,49]. This model will ultimately allow us to integrate the main biophysical and biochemical features that control the development of neuronal growth and the formation of neuronal circuits.

## 5. Conclusions

In this paper, we have introduced a nonlinear dynamical model to analyze neuronal growth on substrates with periodic geometrical patterns. The periodicity of the geometrical patterns on these surfaces is close to the average linear dimension of the growth cone. We have shown that the axonal growth is described by a nonlinear stochastic model with speed-dependent friction function and gaussian white noise. We have demonstrated that this model is in very good agreement with experimental data obtained for the growth cone speed and angular distributions, as well as the axonal mean squared length and diffusion coefficients. Nonlinear dynamics and feedback control account for the complex ways in which neurons respond to environmental stimuli, as well as for the vast array of intricate patterns seen in neuronal development. Our model could be further extended to incorporate various biophysical and biochemical feedback mechanisms that control cellular response and sensitivity to external stimuli. Ultimately, these outcomes will lead to more accurate predictions and a deeper understanding of how neural networks evolve, process information, and adapt to changes. This insight is vital for the development of effective treatments for neurological disorders and the advancement of neuroengineering applications.

## Data Availability

The data presented in this study are available within the manuscript.
